# Supplementary material for: Regulation by the RNA-binding protein Unkempt at its effector interface
Source: Nat Commun. 2024 Apr 11;15:3159. doi: 10.1038/s41467-024-47449-4 (PMC11009413; doi:10.1038/s41467-024-47449-4)
Supplement: Supplementary file 1 — Supplementary Information [file 41467_2024_47449_MOESM1_ESM.pdf]

Regulation by the RNA-binding protein Unkempt at its effector interface

Kriti Shah[1,2,9], Shiyang He[1,2,9], David J. Turner[3,9], Joshua Corbo[3,8], Khadija Rebbani[3], Daniel Dominguez[4], Joseph M. Bateman[5], Sihem Cheloufi[1,2,6], Cátia Igreja[7], Eugene Valkov[3,*], Jernej Murn[1,2,*]

Supplementary Information

9 Supplementary Figures

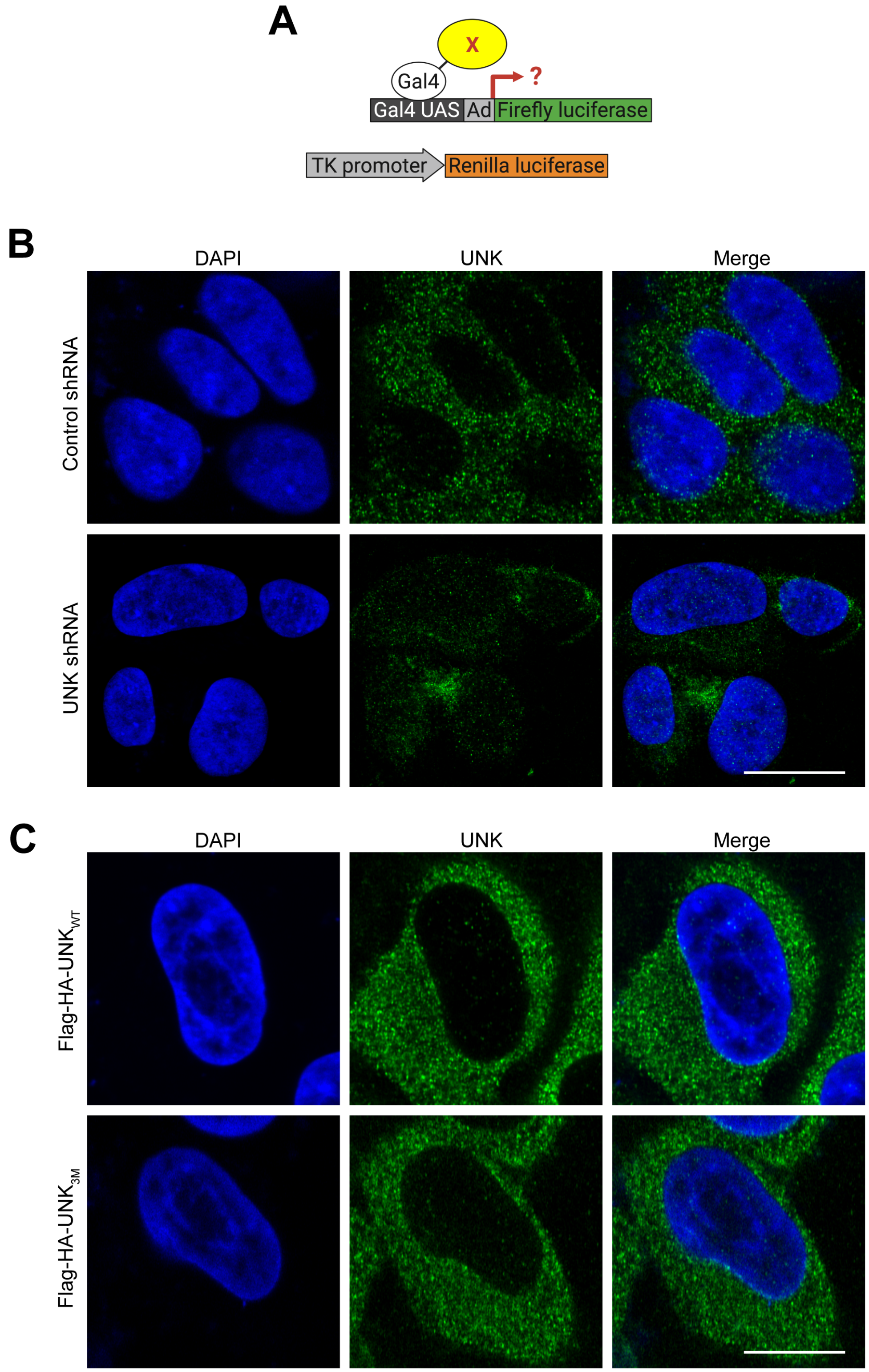

Supplementary Fig. 1 | Transcriptional activity and intracellular localization of Unkempt. **A** Principle of a dual luciferase reporter assay used to detect transcriptional activity of UNK. A tested protein (X) is tagged with the Gal4 DNA-binding domain for recruitment via Gal4

upstream activating sequences (Gal4 UAS) to the adenovirus major late promoter (Ad) driving minimal expression of the firefly luciferase reporter gene. The thymidine kinase (TK) promoter-driven Renilla luciferase serves as an internal control (see Methods). **B** Endogenous UNK detected by immunofluorescence using UNK-specific antibody in SH-SY5Y human neuroblastoma cells stably expressing either shRNA targeting luciferase (Control shRNA, top) or UNK (bottom). Scale bar, 10 µm. **C** Ectopic Flag-HA-tagged UNK_{WT} or UNK_{3M} detected by immunofluorescence using an HA-specific antibody in inducible HeLa cells treated with doxycycline for 24 h. Scale bar, 10 µm. DAPI was used to visualize nuclei. Confocal images shown in **B** and **C** are representative of $n \geq 3$ experiments.

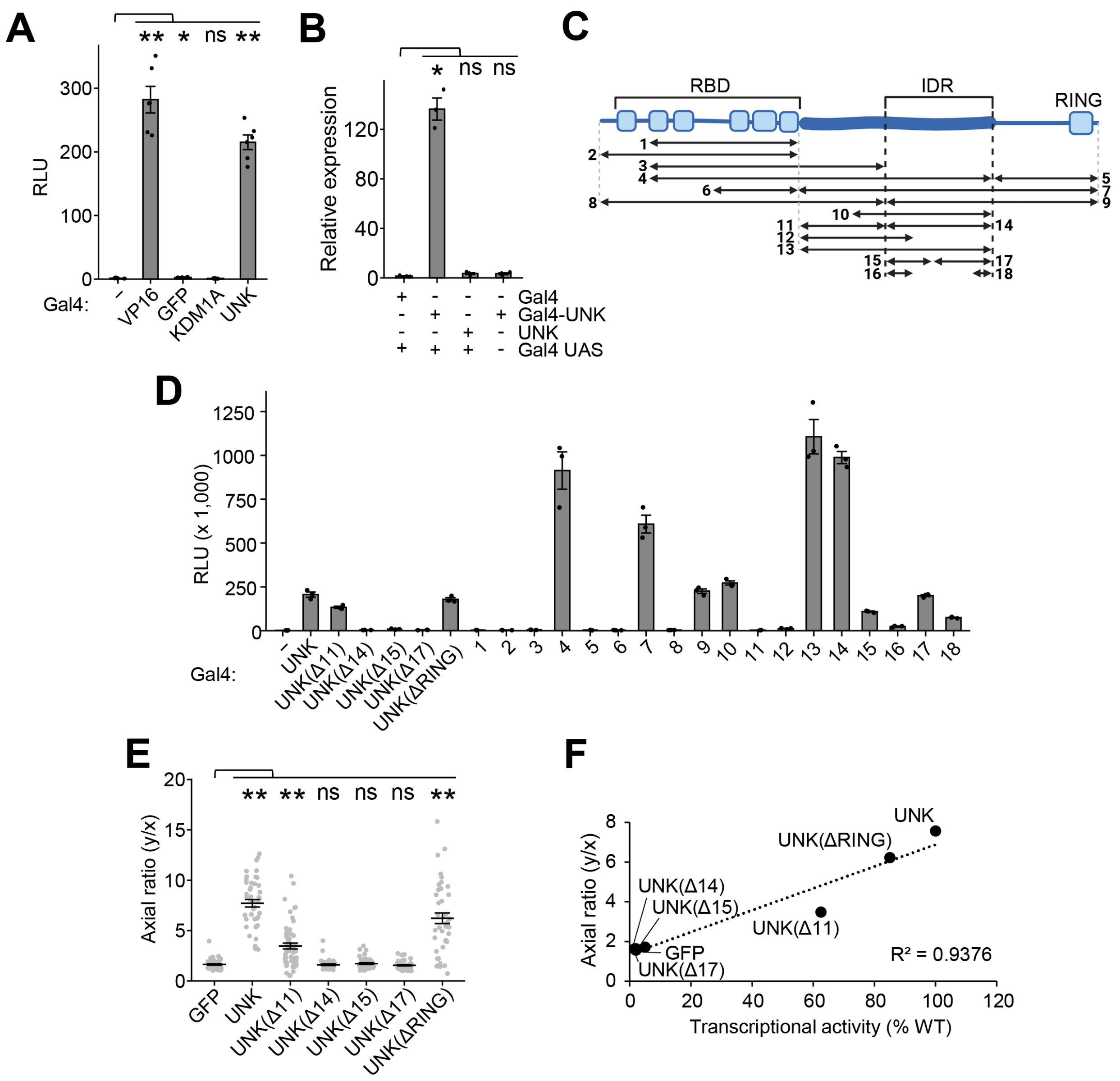

Supplementary Fig. 2 | Morphogenetic and transcriptional activity of Unkempt maps to its IDR.

A Transcriptional activity of UNK measured by a dual luciferase reporter assay at 24 h after transfection of HeLa cells with constructs for expression of the indicated, Gal4 DNA-binding domain (Gal4)-tagged proteins. VP16 transcriptional activator served as a positive control. (–), Gal4 alone; RLU, relative luminescence units (n = 6). **B** Relative quantification by qPCR of firefly luciferase mRNA levels in cells transfected as in **A** with the indicated constructs. Gal4 UAS, Gal4 upstream activating sequences. **C** Domain map of UNK (blue) with the studied segments indicated below by numbers (see also Supplementary Data 1). **D** As in **A**, transcriptional activities of Gal4-tagged deletion mutants UNK(Δ11), UNK(Δ14), UNK(Δ15), UNK(Δ17), UNK(ΔRING), where numbers indicate the segment, defined in panel **C**, deleted from the full-length UNK protein, and Gal4-tagged UNK segments 1 through 18, defined in

defined **C**. **E** Morphologies of cells co-expressing the indicated UNK mutant (see panel **D**) and GFP were quantified by calculating their axial ratios (y/x; Methods)[1] (n = between 42 and 55 GFP-expressing cells per cell line). **F** Correlation of cell morphologies shown in **E** with transcriptional activities of the corresponding Gal4-tagged UNK mutants shown in **D**. Data in **A** (n = 6), **B** (n = 3), **D** (n = 3), and **E** are presented as mean ± SD from biologically independent samples. Statistical significance was determined using Student's t-test with $^{*}p < 0.01$, $^{**}p < 5 \times 10^{-5}$; ns, not significant.

Supplementary Figure 3

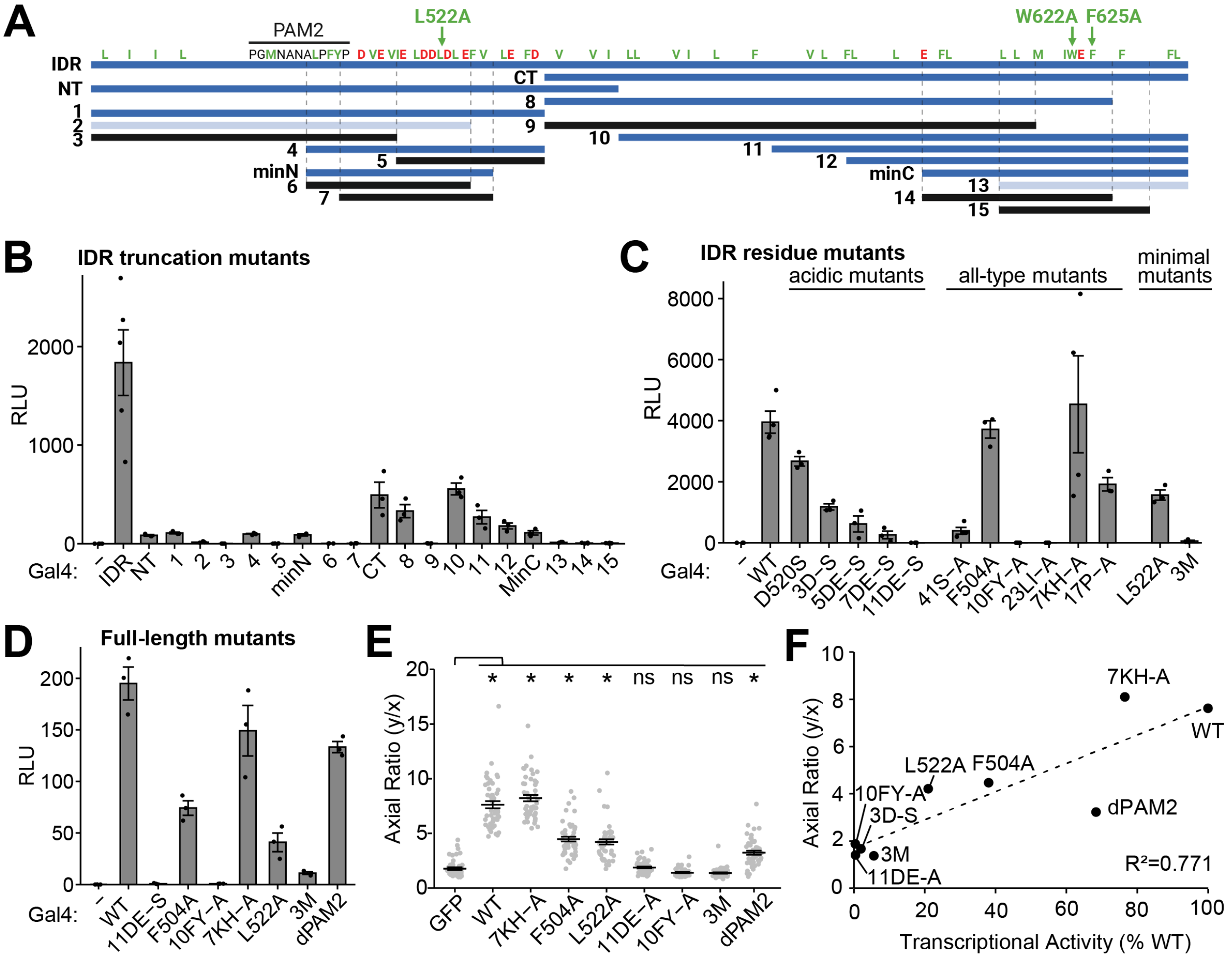

Supplementary Fig. 3 | Identification of key activity-linked features in the IDR of Unkempt. **A** Key truncation mutants of IDR (top blue line) that led to the identification of the smallest transcriptionally active regions, minN and minC. Highly active fragments are in blue, weakly active in light blue, and silent fragments are in black. Positions of hydrophobic residues (I, L, V, M, W, F) and Y are shown in green and acidic residues (D, E) are in red. Green arrows indicate mutations that silence minN (L522A) or minC (W622A and F625A combined). PAM2, the predicted PABPC-binding motif. **B** Dual luciferase reporter assay of Gal4 DNA-binding domain (Gal4)-fusions with IDR or its truncation mutants shown in **A**. (–), Gal4 alone; RLU, relative luminescence units. **C** Contribution of different residues to the transcriptional activity of Gal4-tagged IDR. The indicated mutants were analyzed as in **B**. 'All-type mutants' have all residues of the indicated type mutated to alanines. See also Supplementary Data 1. **D** Contribution of the indicated mutations to the transcriptional activity of the Gal4-tagged full-length UNK, analyzed as in **B**. See also Supplementary Data 1. **E** Morphologies of cells inducibly co-expressing the indicated full-length UNK mutant and GFP at 48 h of incubation with Dox (n = between 47 and 59 GFP-expressing cells per cell line). **F** Correlation of cell morphologies shown in **E** with transcriptional activities shown in **D** for the indicated mutants. Data in **B** (n = 3), **C** (n = 3), **D** (n

= 3), and **E** are presented as mean ± SD from biologically independent samples. Statistical significance was determined using Student's t-test with $^*p < 2 \times 10^{-10}$; ns, not significant.

A

Gene Symbol	MW (kDa)	Total peptide count			
		uninduced	WT	3M	dPAM2
CNOT1	266.77	0	80	0	54
CNOT10	82.26	0	9	0	3
CNOT3	81.82	0	6	0	3
CNOT7	32.72	0	5	0	6
CNOT2	59.7	0	4	0	6
CNOT11	55.18	0	2	0	2
CNOT8	33.52	0	2	0	0
CNOT9	33.61	0	2	0	1
PABPC1	70.63	4	127	105	0
PABPC4	70.74	0	45	32	0

B

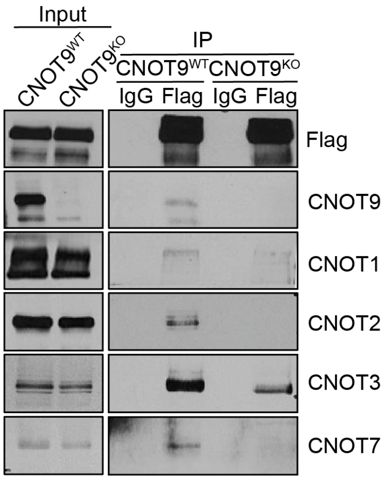

C

```
LIG_PAM2_1: ..[LFP][NS][PIVTAFL].A..(([FY].[PYLF])|(W..)).

                          495         506
                           |           |
H.sapiens   UNKEMPT       PGMNANALPFYP
M.musculus  Unkempt       PGMNANALPFYP
H.sapiens   PAIP1         SKLSVNAPEFYP
H.sapiens   PAIP2         SNLNPNAKEFVP
                          . :. **   * *
```

Supplementary Fig. 4 | Analyses of interactions between Unkempt and its effectors. A Total peptide counts detected by mass spectrometry analysis of tandem affinity-purified protein complexes prepared from uninduced (mock) or UNK_{WT}, UNK_{3M}, or UNK_{dPAM2} - expressing cells (see Methods and Supplementary Data 2). **B** Co-IP of endogenous CCR4-NOT subunits with Flag-HA-tagged UNK_{WT} from *CNOT9*^{WT} or *CNOT9*^{KO} HeLa cells. Precipitated proteins were detected by western blot analysis using the indicated antibodies. Blots are representative of n = 2 biologically independent repeats. **C** Identification of the PAM2-like motif in UNK. A search for either of the two PAM2 motifs annotated in the ELM database (LIG_PAM2_1 and

LIG_PAM2_2; http://elm.eu.org/) identified a 12-residue PAM2-like peptide sequence that deviates from the canonical LIG_PAM2_1 motif (regular expression shown on top) at one position, highlighted in red. PAM2 motifs of PABP-interacting proteins 1 and 2 (PAIP1 and PAIP2) are shown for comparison[2, 3].

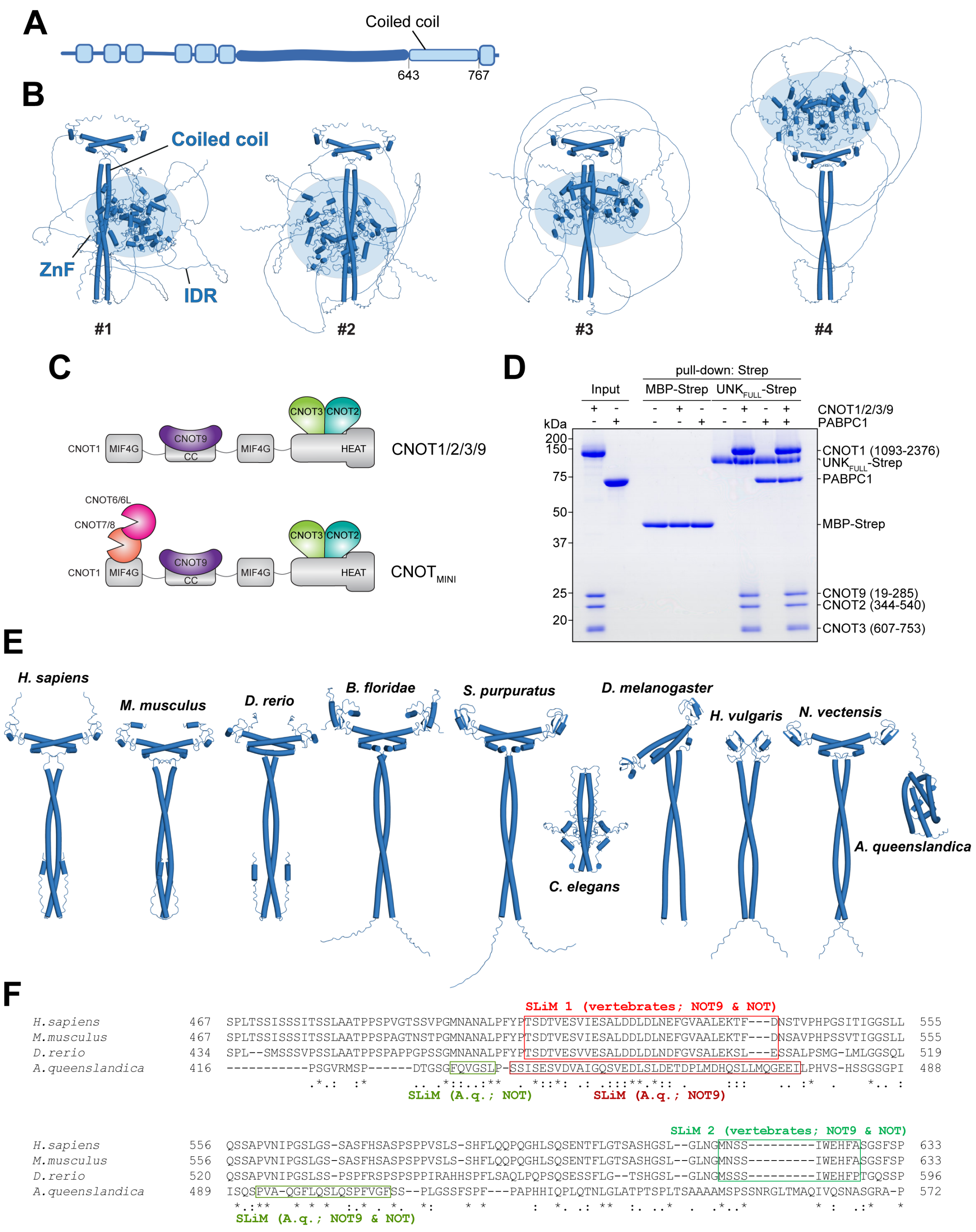

Supplementary Fig. 5 | Structural and functional insights into the Unkempt-effector interface. **A** Map of UNK protein indicating the location of the predicted coiled coil. **B** Four AlphaFold predictions of the homodimer full-length mouse UNK protein showing the central coiled coil stabilizing the dimer with the positioning of the zinc fingers (ZnF, blue sphere) and the unstructured IDR being entirely stochastic with respect to the coil. **C** Schematic representation of the reconstituted CNOT1/2/3/9 (top) and CCR4-NOT$_{MINI}$ (bottom) complexes.

The rationale for using the larger, six-subunit CCR4-NOT_{MINI} complex instead of CNOT1/2/3/9 for the mass photometry experiment shown in Fig. 4D was to avoid signal overlap (the molecular weight of CNOT1/2/3/9 is similar to UNK dimer). **D** Coomassie-stained polyacrylamide gel of an in vitro pull-down assay with MBP-Strep alone or recombinant UNK_{FULL}-Strep after incubation with PABPC1 and/or CNOT1/2/3/9, as indicated (n = 3). **E** A representative AlphaFold prediction of the UNK coiled coil homodimer for each of the shown UNK orthologs (*Homo sapiens*, human; *Mus musculus*, house mouse; *Danio rerio*, zebrafish; *Branchiostoma floridae*, Florida lancelet; *Stronglycentrotus purpuratus*, sea urchin; *Caenorhabditis elegans*, roundworm; *Drosophila melanogaster*, fruit fly; *Hydra vulgaris*, fresh-water polyp; *Nematostella vectensis*, starlet sea anemone; *Amphimedon queenslandica*, sponge). **F** Sequence alignment of IDRs of the indicated UNK orthologs. Locations of SLiMs in contact with CCR4-NOT, as predicted by AlphaFold, are highlighted with red (matching SLiM 1 in mouse UNK) and green (matching SLiM 2 in mouse UNK) rectangles separately for vertebrates and *A. queenslandica* (*A.q.*), which harbors the evolutionarily most distant known UNK ortholog[4,5]. "NOT9" or "NOT" indicates that the SLiM is predicted to only contact one specific region in the respective module and "NOT9 & NOT" indicates repeated SLiM use.

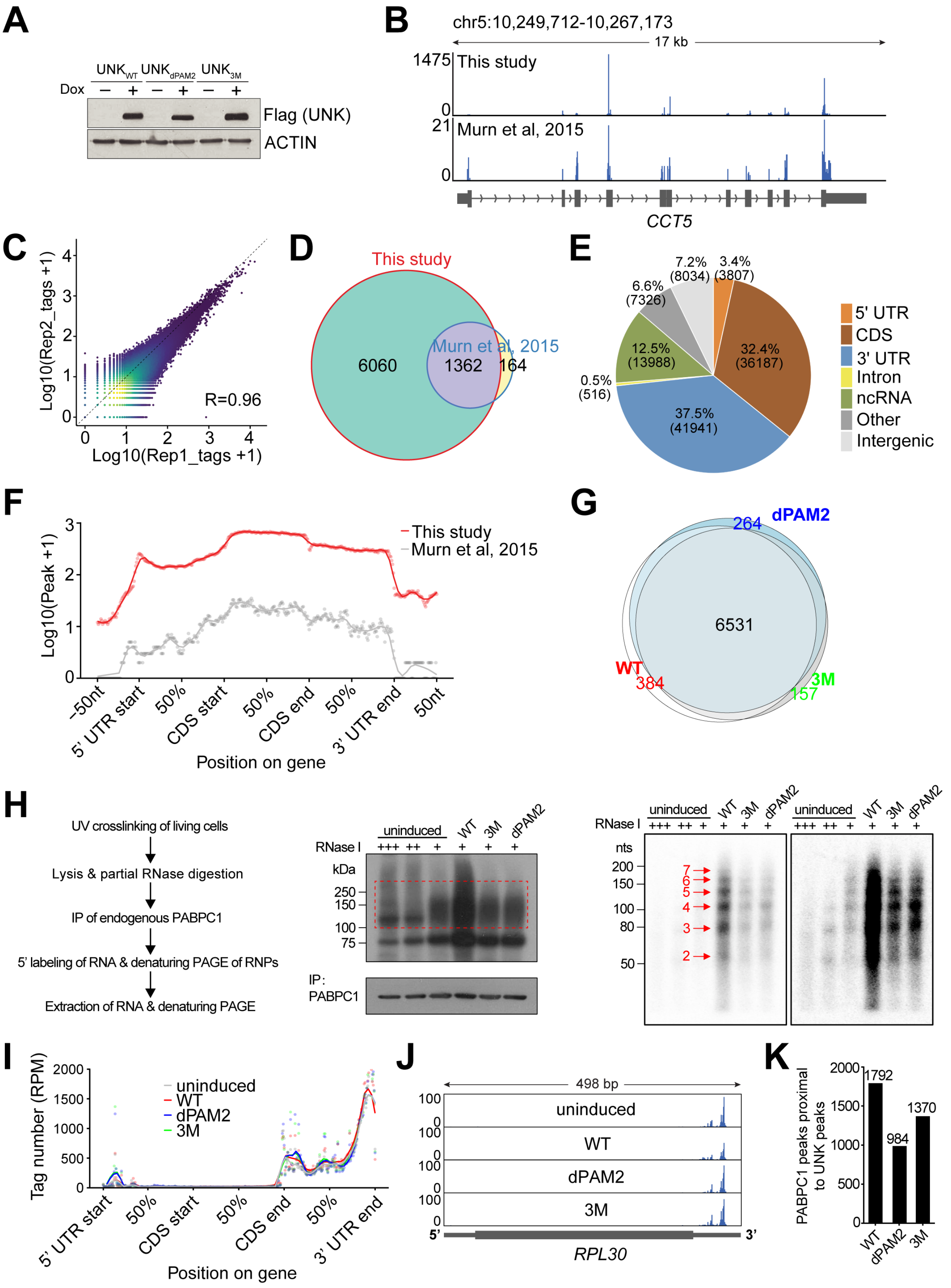
A
UNK_WT UNK_dPAM2 UNK_3M
Dox – + – + – +
Flag (UNK)
ACTIN
B
chr5:10,249,712-10,267,173
17 kb
1475 This study
0
21 Murn et al, 2015
0
CCT5
C
Log10(Rep2_tags +1)
Log10(Rep1_tags +1)
R=0.96
D
This study
Murn et al, 2015
6060
1362
164
E
7.2% (8034)
3.4% (3807)
6.6% (7326)
12.5% (13988)
32.4% (36187)
0.5% (516)
37.5% (41941)
5' UTR
CDS
3' UTR
Intron
ncRNA
Other
Intergenic
F
Log10(Peak +1)
This study
Murn et al, 2015
-50nt
5' UTR start
50%
CDS start
50%
CDS end
50%
3' UTR end
50nt
Position on gene
G
264
dPAM2
6531
WT
384
3M
157
H
UV crosslinking of living cells
Lysis & partial RNase digestion
IP of endogenous PABPC1
5' labeling of RNA & denaturing PAGE of RNPs
Extraction of RNA & denaturing PAGE
uninduced
WT
3M
dPAM2
RNase I
+++ ++ + + + +
kDa
250
150
100
75
IP: PABPC1
nts
200
150
100
80
50
7
6
5
4
3
2
I
Tag number (RPM)
uninduced
WT
dPAM2
3M
5' UTR start
50%
CDS start
50%
CDS end
50%
3' UTR end
Position on gene
J
498 bp
uninduced
WT
dPAM2
3M
5'
3'
RPL30
K
PABPC1 peaks proximal to UNK peaks
1792
984
1370
WT
dPAM2
3M

Supplementary Fig. 6 | RNA binding by UNK_{WT}, UNK_{3M}, UNK_{dPAM2}, and PABPC1. A Western blot analysis of HeLa cells inducibly expressing Flag-HA-tagged UNK_{WT}, UNK_{3M}, or UNK_{dPAM2} at 24 h of treatment with Dox. **B** Genome browser view of the *CCT5* gene locus visualizing reproducibility of UNK_{WT} crosslink events between iCLIP replicates of this (top) and a previous (bottom) study[1]. **C** Density scatterplot comparing crosslink events per peak between replicates 1 and 2 of the UNK_{WT} iCLIP experiment. UNK_{3M} and UNK_{dPAM2} showed similar levels of reproducibility among iCLIP replicates (data now shown). **D** Comparison of mRNA targets of UNK_{WT} in HeLa cells identified by iCLIP in this study and those reported previously[1]. **E** Distribution of UNK_{WT} iCLIP peaks among different RNA biotypes and mRNA segments determined in this study. **F** Metatranscript analyses of the current (red) and previously reported (gray) iCLIP datasets showing the positional frequency of UNK_{WT} binding sites on mRNAs[1]. **G** Comparison of mRNA targets of UNK_{WT}, UNK_{3M}, and UNK_{dPAM2} identified by iCLIP. n = 4 iCLIP replicates were performed for each condition. **H** Analysis of bulk interactions of PABPC1 with poly(A) tails. Labeled, partially RNase-digested PABPC1-bound RNA was extracted from cells expressing or not UNK_{WT}, UNK_{3M}, or UNK_{dPAM2} as outlined in the protocol on the left. Middle panels show autoradiogram of labeled PABPC1 RNP complexes (top) and the corresponding western blot of immunoprecipitated PABPC1 (bottom). Red dashed frame demarcates the area from which RNA was extracted. Two autoradiograms on the right are different exposures of the extracted RNA after resolution by denaturing PAGE. Red arrows point to footprints of 27-nt oligomers (red numbers) protected from RNase, matching the pattern of serial binding of PABPC1 to poly(A) tails[2,6]. Note that the periodic footprint pattern of PABPC1 is not perturbed by expression of UNK but is sensitive to the concentration of RNase I (plus signs). **I**-**K** iCLIP analysis of PABPC1-RNA interactions. **I** Proportional metatranscript analysis of iCLIP data showing the positional frequency of crosslink events for PABPC1 on different segments of mRNA in cells expressing UNK_{WT}, UNK_{3M}, or UNK_{dPAM2}, or in uninduced cells. Data points represent normalized crosslink events summarized over every percent of a given mRNA segment. CDS, coding sequence. **J** Mapping PABPC1 iCLIP data onto UNK target transcripts. iCLIP data from four replicates were summed, normalized, and mapped to *RPL30* mRNA. **K** Occurrence of PABPC1 iCLIP peaks in the vicinity (+/- 20 nts) of UNK_{WT}, UNK_{dPAM2}, or UNK_{3M} iCLIP peaks on mRNA. Note the reduction in the number of observed neighboring peaks upon disrupting UNK–PABPC interactions (UNK_{dPAM2}) and a weaker similar effect upon disrupting UNK–CCR4-NOT interactions (UNK_{3M}). Blots in **A** and **H** are representative of n = 4 biologically independent repeats.

Supplementary Figure 7

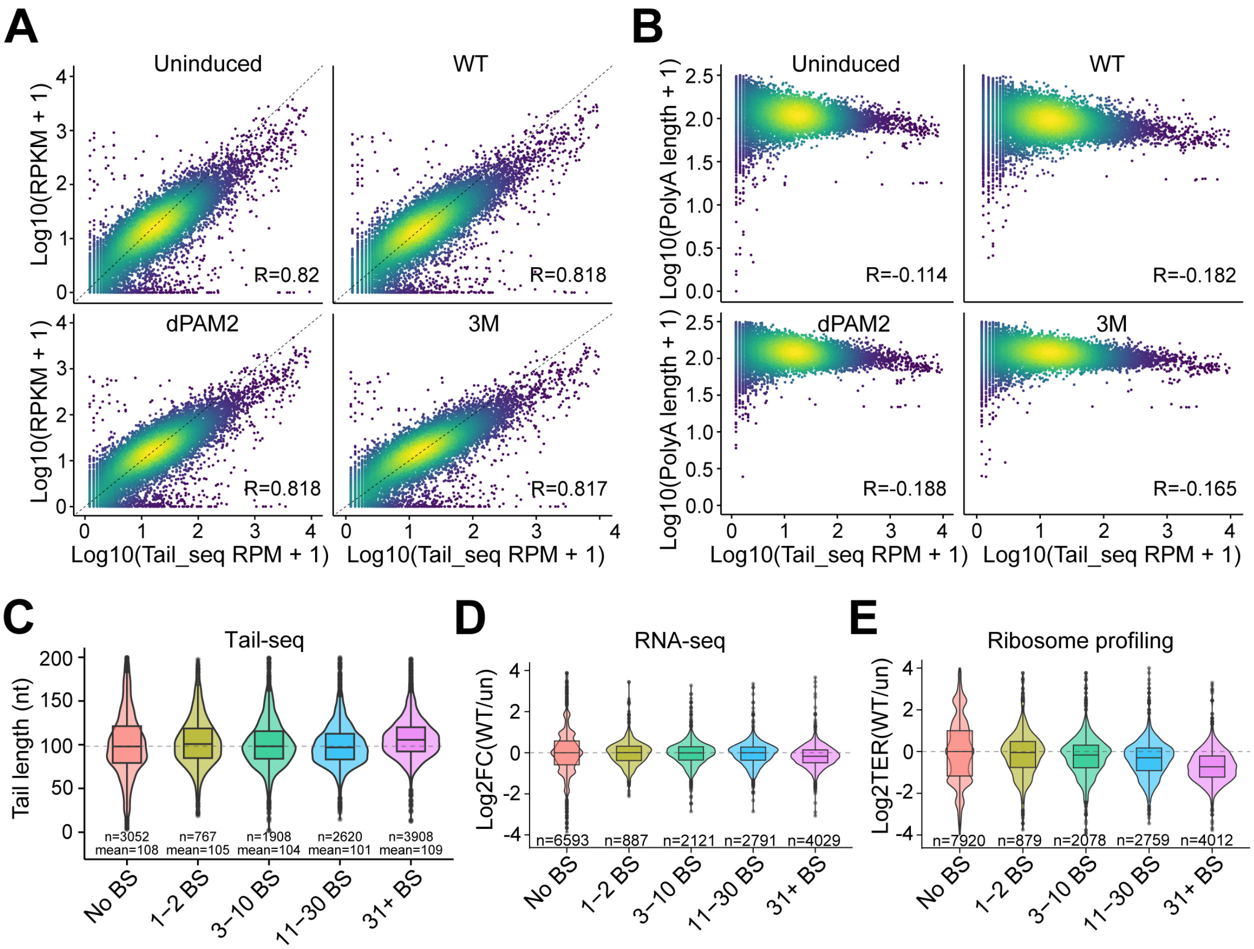

Supplementary Fig. 7 | Impact of Unkempt on mRNA poly(A) tail length. A Scatterplots comparing log10-scaled transcript abundance determined by mRNA-seq (RPKM + 1) and Tail-seq (RPM + 1) analyses of each indicated sample, showing high correlation (n = 2). **B** Scatterplots comparing log10-scaled transcript abundance (RPM + 1) and poly(A) tail length (value + 1), both determined by Tail-seq. Note the slight and expected tendency of highly expressed mRNAs to have relatively short poly(A) tails[7,8,9] (n = 2). **C** Comparison of average poly(A) tail lengths of transcripts binned into groups according to the number of UNK binding sites (BS) per transcript. Data are shown for UNK_{WT}-expressing cells. Similar, minimal changes were observed for uninduced cells and cells expressing UNK_{3M} or UNK_{dPAM2} (data now shown). Numbers of transcripts (n) and mean lengths of their poly(A) tails are indicated for each group of transcripts (n = 2). **D** RNA-seq results for transcripts binned into groups as in **C**, showing changes in steady-state mRNA levels between UNK_{WT}-expressing and uninduced cells (un; n = 3). **E** Ribosome profiling data for transcripts binned into groups as in **C**, showing changes in ribosome occupancy between UNK_{WT}-expressing and uninduced cells (un; n = 2).

Supplementary Figure 8

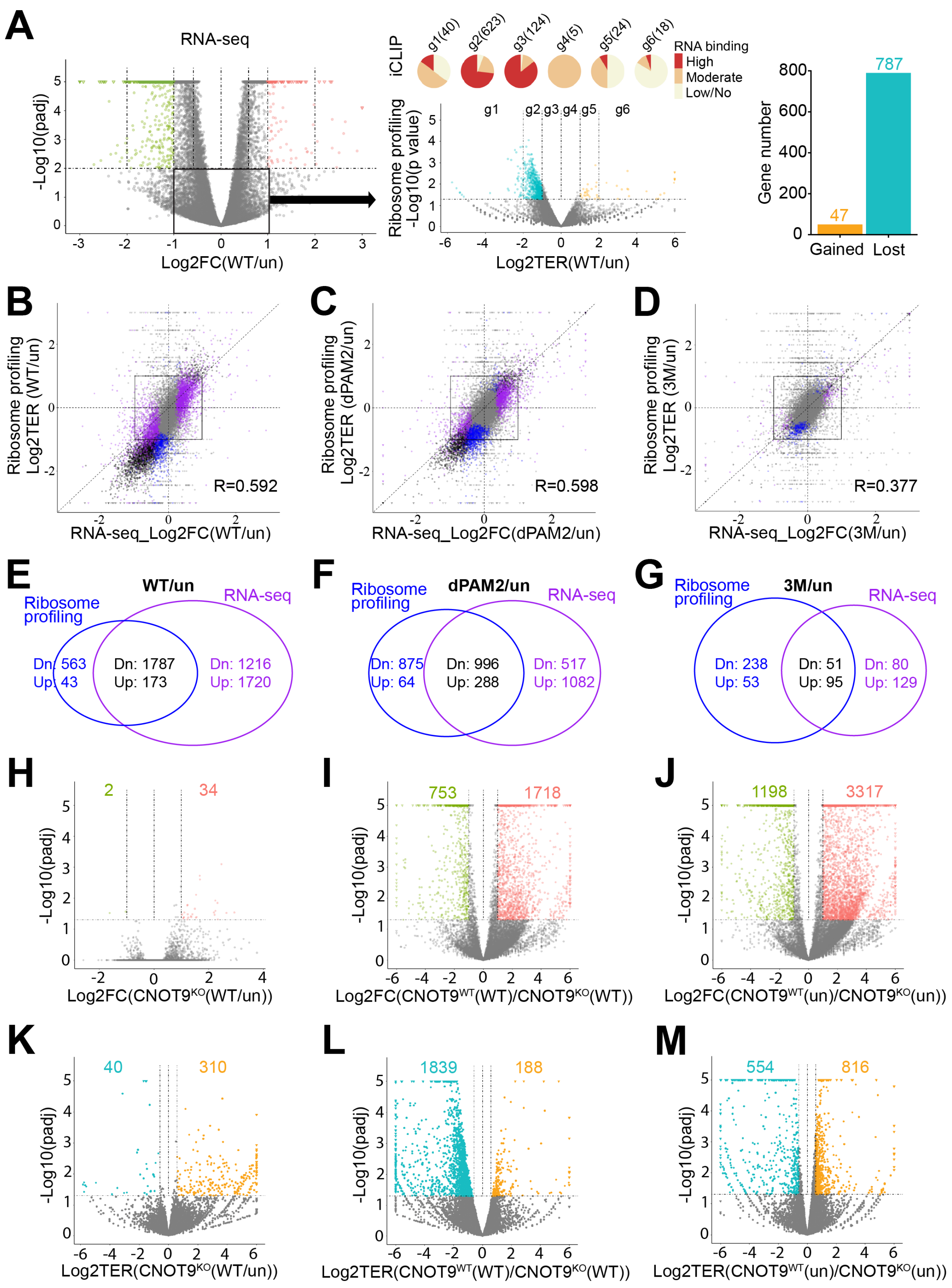

Supplementary Fig. 8 | Reduced translational efficiency mediated by Unkempt. A Translational repression of mRNAs that show little change in abundance upon expression of UNK. mRNAs with small (< 2-fold) and insignificant (adjusted $p > 0.01$) changes in steady-state levels comparing UNK_{WT}-expressing to uninduced cells (group of mRNAs indicated by a black frame in the left plot) were analyzed for changes in ribosome occupancy (middle plot). Pie charts above the middle plot show RNA-binding information and transcript numbers in each group of significantly regulated transcripts (g1-g6), as in Fig. 6E-G. Bar chart on the right indicates total numbers of transcripts with gained or lost ribosome occupancy ($p < 0.05$) ($n = 2$). **B**-**D** Scatterplots comparing differential mRNA abundance (x-axes, RNA-seq data) and differential ribosome occupancy (y-axes, ribosome profiling data) between uninduced cells (un) and cells expressing UNK_{WT} (**B**), UNK_{dPAM2} (**C**), or UNK_{3M} (**D**). Transcripts highlighted in purple, blue, or black are significantly regulated on the mRNA level only (p adj. < 0.05), on the level of ribosome occupancy only ($p < 0.05$), or on both levels (RNA-seq p adj. < 0.05 and ribosome profiling p value < 0.05), respectively. FC, fold change; TER, translational efficiency ratio. See also Fig. 6A-G. **E**-**G** Proportional Venn diagrams showing numbers of significantly regulated transcripts, as highlighted in **B**-**D**, in the overlap between RNA-seq and ribosome profiling data for the indicated comparisons. Dn and Up, decrease and increase in mRNA level or ribosome occupancy, respectively; un, uninduced cells. **H**-**M** Reliance of translational repression by UNK on CNOT9. Three pairs of samples are compared by RNA-seq (**H**-**J**) and ribosome profiling (**K**-**M**) to document this reliance as well as the effect of CNOT9 alone: (**H**, **K**) CNOT9 KO cells ($CNOT9^{KO}$) expressing or not UNK_{WT}, (**I**, **L**) CNOT9 WT and CNOT9 KO cells expressing UNK_{WT}, and (**J**, **M**) uninduced (un) CNOT9 WT ($CNOT9^{WT}$) and $CNOT9^{KO}$ cells. Significantly regulated transcripts with twofold or larger changes in abundance (RNA-seq) or ribosome occupancy (ribosome profiling) are highlighted in color ($n = 3$ for RNA-seq; $n = 2$ for ribosome profiling).

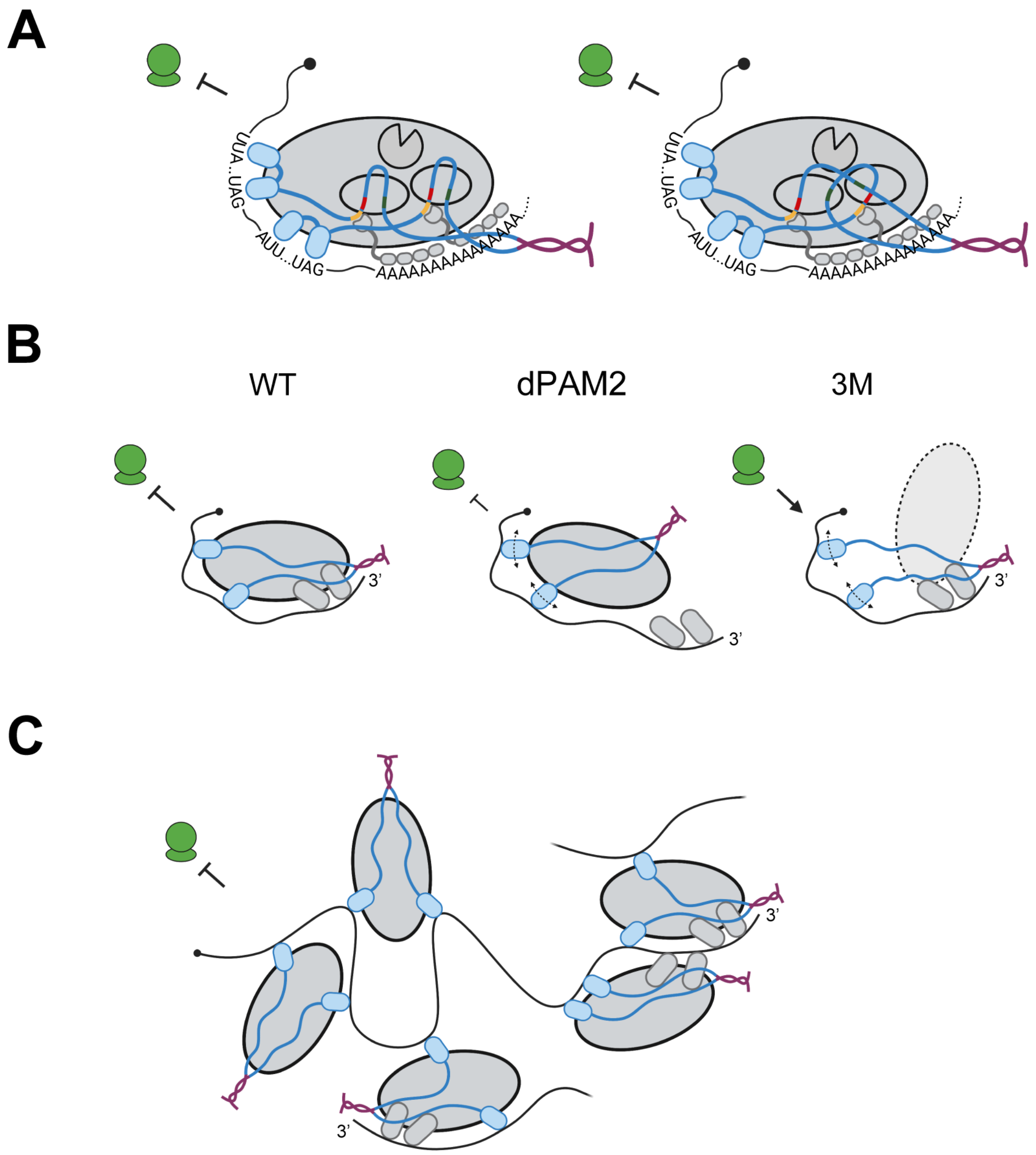

Supplementary Fig. 9 | PABPC1-RNA interactions and models of Unkempt-effector interface. **A**-**C** Models of UNK-effector interface. **A** Two possible modes of interaction between the IDR-embedded SLiMs of UNK and the NOT9 and NOT modules of CCR4-NOT. See also Fig. 7. **B** Models illustrating how disrupting the interactions between UNK and PABPC (UNK_{dPAM2}) or CCR4-NOT (UNK_{3M}) might affect RNP organization and function. Curved dashed lines with arrowheads on each end indicate compromised RNA sequence recognition by UNK. Inhibitory arrows pointing to the ribosome (green shapes) or the arrow pointing to the RNP indicate translational repression or lack thereof, respectively. **C** A larger RNP particle formed via association of multiple copies of the UNK dimer–CCR4-NOT–PABPC complex with mRNA. Created with BioRender.com.

SUPPLEMENTARY REFERENCES

1. Murn J*, et al.* Control of a neuronal morphology program by an RNA-binding zinc finger protein, Unkempt. *Genes Dev* **29**, 501-512 (2015).

2. Khaleghpour K*, et al.* Translational repression by a novel partner of human poly(A) binding protein, Paip2. *Mol Cell* **7**, 205-216 (2001).

3. Craig AW, Haghighat A, Yu AT, Sonenberg N. Interaction of polyadenylate-binding protein with the eIF4G homologue PAIP enhances translation. *Nature* **392**, 520-523 (1998).

4. Murn J, Teplova M, Zarnack K, Shi Y, Patel DJ. Recognition of distinct RNA motifs by the clustered CCCH zinc fingers of neuronal protein Unkempt. *Nat Struct Mol Biol* **23**, 16-23 (2016).

5. Srivastava M*, et al.* The Amphimedon queenslandica genome and the evolution of animal complexity. *Nature* **466**, 720-726 (2010).

6. Yi H, Park J, Ha M, Lim J, Chang H, Kim VN. PABP Cooperates with the CCR4-NOT Complex to Promote mRNA Deadenylation and Block Precocious Decay. *Mol Cell* **70**, 1081-1088 e1085 (2018).

7. Lima SA*, et al.* Short poly(A) tails are a conserved feature of highly expressed genes. *Nat Struct Mol Biol* **24**, 1057-1063 (2017).

8. Subtelny AO, Eichhorn SW, Chen GR, Sive H, Bartel DP. Poly(A)-tail profiling reveals an embryonic switch in translational control. *Nature* **508**, 66-71 (2014).

9. Workman RE*, et al.* Nanopore native RNA sequencing of a human poly(A) transcriptome. *Nat Methods* **16**, 1297-1305 (2019).
