# Supplementary material for: Regulation by the RNA-binding protein Unkempt at its effector interface
Source: Nat Commun. 2024 Apr 11;15:3159. doi: 10.1038/s41467-024-47449-4 (PMC11009413; doi:10.1038/s41467-024-47449-4)
Supplement: Supplementary file 3 — Description of Additional Supplementary Information [file 41467_2024_47449_MOESM3_ESM.pdf]

Description of Additional Supplementary Information

Supplementary Data 1. List of UNK mutants used in this study.

Supplementary Data 2. Analyses of UNKWT, UNK3M, and UNKdPAM2 protein complexes by mass spectrometry.

Supplementary Data 3. iCLIP targets of UNKWT, UNK3M, and UNKdPAM2 in HeLa cells.

Supplementary Data 4. PABPC1 iCLIP targets in uninduced HeLa cells and in HeLa cells expressing UNKWT, UNK3M, or UNKdPAM2.

Supplementary Data 5. RNA-seq analyses of uninduced HeLa cells and of HeLa cells expressing UNKWT, UNK3M, or UNKdPAM2. Statistical significance was calculated using the Wald test (p values) with Benjamini-Hochberg adjustment for multiple comparisons (p adj).

Supplementary Data 6. Poly(A) tail length analyses of uninduced HeLa cells and of HeLa cells expressing UNKWT, UNK3M, or UNKdPAM2.

Supplementary Data 7. Ribosome profiling analyses of uninduced HeLa cells and of HeLa cells expressing UNKWT, UNK3M, or UNKdPAM2. Statistical significance was calculated using the Wald test (p values) with Benjamini-Hochberg adjustment for multiple comparisons (p adj).
