# Supplementary material for: Regulation by the RNA-binding protein Unkempt at its effector interface
Source: Nat Commun. 2024 Apr 11;15:3159. doi: 10.1038/s41467-024-47449-4 (PMC11009413; doi:10.1038/s41467-024-47449-4)
Supplement: Supplementary file 11 — Reporting Summary [file 41467_2024_47449_MOESM11_ESM.pdf]

nature portfolio

Corresponding author(s): Jernej Murn, Eugene Valkov

Last updated by author(s): Feb 6, 2024

Reporting Summary

Nature Portfolio wishes to improve the reproducibility of the work that we publish. This form provides structure for consistency and transparency in reporting. For further information on Nature Portfolio policies, see our Editorial Policies and the Editorial Policy Checklist.

Statistics

For all statistical analyses, confirm that the following items are present in the figure legend, table legend, main text, or Methods section.

n/a	Confirmed	
☐	☒	The exact sample size (*n*) for each experimental group/condition, given as a discrete number and unit of measurement
☐	☒	A statement on whether measurements were taken from distinct samples or whether the same sample was measured repeatedly
☐	☒	The statistical test(s) used AND whether they are one- or two-sided<br>*Only common tests should be described solely by name; describe more complex techniques in the Methods section.*
☒	☐	A description of all covariates tested
☐	☒	A description of any assumptions or corrections, such as tests of normality and adjustment for multiple comparisons
☐	☒	A full description of the statistical parameters including central tendency (e.g. means) or other basic estimates (e.g. regression coefficient) AND variation (e.g. standard deviation) or associated estimates of uncertainty (e.g. confidence intervals)
☐	☒	For null hypothesis testing, the test statistic (e.g. *F*, *t*, *r*) with confidence intervals, effect sizes, degrees of freedom and *P* value noted<br>*Give P values as exact values whenever suitable.*
☒	☐	For Bayesian analysis, information on the choice of priors and Markov chain Monte Carlo settings
☒	☐	For hierarchical and complex designs, identification of the appropriate level for tests and full reporting of outcomes
☒	☐	Estimates of effect sizes (e.g. Cohen's *d*, Pearson's *r*), indicating how they were calculated

Our web collection on statistics for biologists contains articles on many of the points above.

Software and code

Policy information about availability of computer code

Data collection	Microscopy data was collected with LAS X (Leica) and ZEN (Zeiss) software. Mass photometry data was collected with AcquireMP (version 2023 R1.1) software. RT-qPCR data was collected with CFX Maestro (v2.2). Sequencing data was collected by Illumina Real-Time Analysis software. Github web-link to the custom code developed in this study: https://github.com/Shiyang-He/Unkempt-Project-data-analysis/.
Data analysis	Data analysis was conducted with GraphPad Prism v9.2.0 or with R v3.6.2. Mass photometry data analysis was performed with DiscoverMP (version 2023 R1.2) software.

For manuscripts utilizing custom algorithms or software that are central to the research but not yet described in published literature, software must be made available to editors and reviewers. We strongly encourage code deposition in a community repository (e.g. GitHub). See the Nature Portfolio guidelines for submitting code & software for further information.

Data

Policy information about availability of data

All manuscripts must include a data availability statement. This statement should provide the following information, where applicable:

- Accession codes, unique identifiers, or web links for publicly available datasets
- A description of any restrictions on data availability
- For clinical datasets or third party data, please ensure that the statement adheres to our policy

The high-throughput sequencing data generated in this study have been deposited in the Gene Expression Omnibus (GEO) database under accession code GSE240571 (https://www.ncbi.nlm.nih.gov/geo/query/acc.cgi?acc=GSE240571). The mass spectrometry proteomics data have been deposited in the ProteomeXchange Consortium via the PRIDE86 partner repository with the dataset identifier PXD050601 (https://proteomecentral.proteomexchange.org/cgi/GetDataset?ID=PXD050601).

Research involving human participants, their data, or biological material

Policy information about studies with human participants or human data. See also policy information about sex, gender identity/presentation, and sexual orientation and race, ethnicity and racism.

Reporting on sex and gender	N/A
Reporting on race, ethnicity, or other socially relevant groupings	N/A
Population characteristics	N/A
Recruitment	N/A
Ethics oversight	N/A

Note that full information on the approval of the study protocol must also be provided in the manuscript.

Field-specific reporting

Please select the one below that is the best fit for your research. If you are not sure, read the appropriate sections before making your selection.

☒ Life sciences ☐ Behavioural & social sciences ☐ Ecological, evolutionary & environmental sciences

For a reference copy of the document with all sections, see nature.com/documents/nr-reporting-summary-flat.pdf

Life sciences study design

All studies must disclose on these points even when the disclosure is negative.

Sample size	There was no specific statistical method used to determine sample size. We used sample sizes that yielded sufficient statistical power in previous studies from our labs such as in Murn et al., Genes Dev (2015), Poetz et al, Nat Commun (2021), Franklin et al., Nat Commun (2022)
Data exclusions	Data were only excluded for failed experiments resulting from technical issues.
Replication	Experiments were repeated in triplicates to confirm experimental reproducibility. Multiple biological samples were tested to confirm biological reproducibility. All results reported in the manuscript have been reproduced in independent experiments.
Randomization	Cell samples were randomly allocated into experimental groups.
Blinding	The investigators were not blinded to group allocation during experiments. Blinding was not possible for the in vitro and in vivo studies as these experiment were performed by individual investigators who were aware of the experimental groups. Blinding the investigator is also not relevant for bioinformatic analyses of large datasets as they are performed using computational algorithms.

Reporting for specific materials, systems and methods

We require information from authors about some types of materials, experimental systems and methods used in many studies. Here, indicate whether each material, system or method listed is relevant to your study. If you are not sure if a list item applies to your research, read the appropriate section before selecting a response.

Materials & experimental systems

n/a	Involved in the study
☐	☒ Antibodies
☐	☒ Eukaryotic cell lines
☒	☐ Palaeontology and archaeology
☒	☐ Animals and other organisms
☒	☐ Clinical data
☒	☐ Dual use research of concern
☒	☐ Plants

Methods

n/a	Involved in the study
☒	☐ ChIP-seq
☒	☐ Flow cytometry
☒	☐ MRI-based neuroimaging

Antibodies

Antibodies used

The primary antibodies were used at the following dilutions: 1:1000 for western blotting (except anti-β-Actin-peroxidase antibody, which was used at 1:20,000), 1:250 for immunofluorescence, 1:50 for immunoprecipitation experiments. All commercially-available antibodies were from were multiple lots.
Anti-UNK antibody produced in rabbit, Millipore Sigma, Cat#HPA023636; RRID:AB_10601774
Monoclonal ANTI-FLAG M2 antibody produced in mouse, Millipore Sigma, Cat#F1804; RRID:AB_262044
CNOT1 Polyclonal antibody, Proteintech Cat#14276-1-AP; RRID:AB_10888627
CNOT2 (D8Z8P) Rabbit mAb, Cell Signaling Technology Cat#34214; RRID:AB_2799049
CNOT3 Polyclonal antibody, Proteintech Cat#11135-1-AP; RRID:AB_2229682
Rabbit polyclonal anti-CNOT7 antibody, gift of A.B. Shyu (Yamashita, A. et al. Concerted action of poly(A) nucleases and decapping enzyme in mammalian mRNA turnover. Nat Struct Mol Biol 12, 1054-1063.)
Anti-RQCD1 (CNOT9) antibody, Fine Test, Cat#FNab07487
Anti-PABP antibody, Abcam, Cat#ab21060; RRID:AB_777008
Purified anti-HA.11 Epitope Tag Antibody, BioLegend Cat#901502; RRID:AB_2565007
Normal mouse IgG, Santa Cruz Biotechnology Cat#sc-2025; RRID:AB_737182
Anti-β-Actin–Peroxidase antibody, Mouse monoclonal, Millipore Sigma Cat#A3854; RRID:AB_262011
Anti-Mouse IgG (Fc specific)–Peroxidase antibody produced in goat, Millipore Sigma Cat#AP124P; RRID:AB_90456
Anti-Rabbit IgG (whole molecule)–Peroxidase antibody produced in goat, Millipore Sigma Cat#AP307P; RRID:AB_92641
Goat anti-Mouse IgG (H+L) Highly Cross-Adsorbed Secondary Antibody, Alexa Fluor 488, Thermo Fisher Scientific Cat#11029; RRID:AB_2534088
Goat anti-Rabbit IgG (H+L) Highly Cross-Adsorbed Secondary Antibody, Alexa Fluor 488, Thermo Fisher Scientific Cat#A-11034; RRID:AB_2576217

Validation

We validated UNK, FLAG, CNOT1, CNOT2, CNOT3, CNOT7, RQCD1 (CNOT9), and HA.11 antibodies by western blotting using knockdown, knockout, or Flag-HA-UNK expressing HeLa cells. We also validated UNK, FLAG, and HA.11 antibodies by immunofluorescence in knockdown or Flag-HA-UNK expressing SH-SY5Y or HeLa cells.
The PABP antibody was validated by the manufacturer (Abcam) and is cited for use in western blotting, immunofluorescence, and immunoprecipitation (including CLIP) by 120 references. This information can be found at the following website: https://www.abcam.com/products/primary-antibodies/pabp-antibody-ab21060.html?productWallTab=Abreviews

Eukaryotic cell lines

Policy information about cell lines and Sex and Gender in Research

Cell line source(s)	HeLa cells, ATCC (CCL-2); SH-SY5Y cells, ATCC (CRL-2266); 293T cells, ATCC (CRL-3216)
Authentication	Cells were authenticated by ATCC using STR profiling.
Mycoplasma contamination	Cell lines routinely tested negative for mycoplasma.
Commonly misidentified lines (See ICLAC register)	None of cell lines used in this study are listed in the ICLAC register.

Plants

Seed stocks	*Report on the source of all seed stocks or other plant material used. If applicable, state the seed stock centre and catalogue number. If plant specimens were collected from the field, describe the collection location, date and sampling procedures.*
Novel plant genotypes	*Describe the methods by which all novel plant genotypes were produced. This includes those generated by transgenic approaches, gene editing, chemical/radiation-based mutagenesis and hybridization. For transgenic lines, describe the transformation method, the number of independent lines analyzed and the generation upon which experiments were performed. For gene-edited lines, describe the editor used, the endogenous sequence targeted for editing, the targeting guide RNA sequence (if applicable) and how the editor was applied.*
Authentication	*Describe any authentication procedures for each seed stock used or novel genotype generated. Describe any experiments used to assess the effect of a mutation and, where applicable, how potential secondary effects (e.g. second site T-DNA insertions, mosiacism, off-target gene editing) were examined.*
